# Slaughter performance and meat quality of Begait breed lambs fattened under different diets

**DOI:** 10.1016/j.heliyon.2021.e06935

**Published:** 2021-05-03

**Authors:** Kahsu Atsbha, Tikabo Gebremariam, Teferi Aregawi

**Affiliations:** a Humera Agricultural Research Center, Tigray Agricultural Research Institute, P.O.Box: 62, Humera, Ethiopia; b Department of Animal, Rangelands and Wildlife Sciences, Mekelle University, P.O.Box: 231, Mekelle, Ethiopia; c Tigray Agricultural Research Institute, P.O.Box: 492, Mekelle, Ethiopia

**Keywords:** Carcass traits, Lamb feeding, Meat quality, Sheep

## Abstract

This study was conducted to evaluate slaughter performance and meat quality of Begait sheep breed reared under different feeding options. Thirty intact fattening lambs (32.81 ± 2.39 kg) were used in randomized completely block design with three dietary treatments in ten replicates for a 90 day feeding trial. Dietary treatments constituted grass hay fed *ad libitum* to all treatments plus supplemented with mixed diet of 48% wheat bran, 15% molasses, 35% cotton seed cake, 1% salt and 1% limestone (T1); 43% wheat bran, 20% sorghum grain, 35% noug seed cake, 1% salt and 1% limestone (T2) and 47% wheat bran, 16% molasses, 35% sesame seed cake, 1% salt and 1% limestone (T3). At the end of the experimental period, six lambs randomly chosen from each treatment were slaughtered to determine the carcass traits and meat quality. Results showed that most carcass and meat traits were affected by diets. Average daily gain (ADG) was higher (*P* < 0.001) for T3 (158 g/day) than T2 (120 g/day) and T1 (118 g/day). Hot carcass weight (HCW) was higher (*P* < 0.001) for T3 (19.50 kg) than T2 (17.43 kg) and T1 (17.20 kg). Meat pH (5.43–5.56) and drip loss (3.03–3.23%) were similar (P > 0.05) among all treatments. Meat from animals in T3 (33.97 L∗) was lighter (*P* < 0.001) than T2 (30.75 L∗) and T1 (29.43 L∗). Shear force and cooking loss were greater (*P* < 0.05) for T1 (42.6 N and 26.12%) than T2 (40.4 N and 24.39%) and T3 (40.7 N and 24.18%). No variation was seen in meat moisture, protein and ash contents (*P* > 0.05) while intramuscular fat was higher in T3 (4.18%) than T2 (3.87%) and T1 (3.79%) (*P* < 0.01). The study indicated that carcass traits and meat quality could be modulated through dietary manipulation.

## 1 Introduction

In almost all parts of Ethiopia, the routine livelihood activity of smallholder farmers depends on agricultural practices. The livestock sector plays a considerable role in poverty reduction, national income growth, exports and foreign exchange earnings, in achieving better food security and contribute to climate mitigation and adaptation (AGP-LMD, 2013; Shapiro et al., 2015). About 31.30 million sheep are estimated in the country (CSA, 2018). Despite the existence of large number and diversified types of sheep in the country, its strategic location, and the adaptation of the importers to the taste of sheep meat, the contribution of sheep to national economy is extremely low (AGP-LMD, 2013). Inadequate nutrition has been recognized as a main constraint to the low productivity of Ethiopian livestock (FAO, 1993; 1998; Mengistu, 2006; Tolera, 2012; Assefa et al., 2010; Tegegne and Assefa, 2010; IPMS, 2012).

The major feed bases of sheep in Ethiopia are natural pasture, grass hay and crop residues (Mengistu, 2006; Tolera, 2012; Yami, 2008; Tegegne and Assefa, 2010). Nutritional content of these feed bases (Bediye and Sileshi, 1989) is less than the optimum requirement for maintenance (Van Soest, 1994; McDonald et al., 2002) leave alone supporting growth and milk production (NRC, 1985). Deficiencies of nutrient that reduce the activities of rumen microorganisms are responsible to decrease feed intake. The most common is protein or nitrogen deficiency, which may be corrected by supplementation with rumen-degradable protein or with a simple source of nitrogen. Subsequently, Ethiopian sheep are sold in the market with poor body condition (Van Soest, 1994; McDonald et al., 2002). For instance, the averages hot carcass yields of Ethiopian sheep (10 kg) is the lowest relative to all countries and the world average (Getachew and Mohamadou, 2014). This has also led to low annual per capita domestic meat consumption at household and national levels (<10kg) (FAO, 1993) and the economic return of the small holder farmers and the exporting value for the country.

There is a need to significantly enhance livestock production and productivity to meet the ever-increasing demand for livestock products (MoA, 2014–unpublished). This calls for planned supplementation of the roughage-based livestock to overcome the inadequate problem of feed and improve productivity through formulating an optimum balanced diet. In the tropics, crude protein has been recognized as the most limiting nutrient during dry seasons. Supplementation of dietary is known to enhance intake by increasing the supply of nitrogen to the rumen microbes. This has a positive effect in increasing rumen microbial population and efficiency, thus allowing them to enhance the degree of breakdown of the digesta. As the degree of digesta breakdown increases, feed intake also increased (Van Soest, 1994; McDonald et al., 2002).

In the study area, the most available and affordable plant protein source ingredients for smallholder livestock producers are sesame seed cake, cotton seed cake and noug seed cake. In spite of the availability of these agro-industrial by-products in the study area, smallholder farmers are not effectively utilized by for feeding. This is mainly due to lack of information and practice. However, the use of these by-products is a potential option through which the productivity of animals can be enhanced. Thus, supplementing sheep with protein source concentrate which are less competitive in human food system is commendable.

Begait sheep breed is one of the identified indigenous Ethiopian sheep breeds located in the western zone of Tigray with promising production and reproduction potential, resulting in high litter size, early maturity, short lambing interval, and high milk yield (Tsigab et al., 2018). Despite the potential of this breed, very limited research works have been published on the fattening performance and carcass characteristics by different scholars (Yirdaw et al., 2017; Gebrekidan et al., 2019; Haymanot et al., 2019). However, there is no any published information regarding the meat quality of this breed. With this mindful, optimum carcass production performance and meat quality traits of Begait sheep breeds need to be studied, and information should be made available for future improvement endeavors. With the intention to address the existing serious lack of information, this study was intended to evaluate slaughter performance and meat quality traits of Begait sheep breed fattened under different feeding regimes using locally available feed resources and thus to determine the optimum feeding option.

## 2 Materials and methods

### 2.1 Experimental area

The experiment was conducted at the small ruminant research unit of Humera Agricultural Research Center, Northern Ethiopia. The experimental site is located at 14° 15′ N latitude and 36° 37′ E longitude at an altitude of 608.9 m above sea level. The area receives an annual rainfall ranging from 400 to 650 mm. The annual temperature of the area ranges from 22.2 to 42 °C. The climatic condition of the area is hot and semi-arid with a uni-modal rainfall pattern (Tadesse and Abay, 2011).

#### 2.1.1 Animal care

The experiment was approved (19641ET-27/2020) by the Ethics Committee of the Tigray Agricultural Research Institute following guidelines of the European Union directive number 2010/63/EU (2010) regarding the care and use of animals for experimental and scientific purposes.

### 2.2 Animals, diets and experimental design

Thirty vaccinated and dewormed yearling intact growing male Begait lambs (32.81 ± 2.39kg) were sourced from near sheep farm to the study area. The lambs were kept for 90 days, which were preceded by 15 days of acclimation to the new facilities and diets with the considering of animal welfare.

The animals were blocked according to their initial body weight which was determined by taking the mean of two consecutive weightings after overnight fasting for water and feed. They were grouped into ten blocks of three animals each based on initial body weight. The three treatment diets were randomly assigned to each experimental animal. Each animal within the block got an equal chance of receiving one of the treatment diets. The experimental diets were consisted of grass hay fed *ad libitum* common to all groups and supplemented with a mixed diet of 48% wheat bran (wb), 15% molasses, 35% cottonseed cake (csc), 1% salt and 1% limestone (T1); 43% wb, 20% sorghum grain, 35% noug seed cake(nsc), 1% salt and 1% limestone (T2) and 47% wb, 16% molasses, 35% sesame seed cake (ssc), 1% salt and 1% limestone (T3). The experimental treatments were chosen for their availability and potential utilization in the study area. The diet was offered with a grass hay and concentrate, targeting for a weight gain of 200 g/day following the NRC (2007) and based on data from a chemical analysis of roughage and concentrates performed previously during the acclimation period. The chemical composition of feed ingredients and treatment diets are shown in Table 1. The crude protein (CP) contents of mixture diets consumed by sheep groups were 20.25%, 20.66%, and 20.88% for T1, T2, and T3, respectively. Likewise, the sheep consumed diets containing 68.97% (T1), 70.66% (T2), and 76.03% (T3) total digestible nutrient (TDN), which measures energy value. The supplement feed was offered in two equal portions twice daily (09:00 am and 02:00 pm). All lambs were weighed on the same dates and times (before morning feeding) every ten days throughout the experimental days. Lambs were kept in individual pens and allowed *ad libitum* access to natural grass hay and free water.

**Table 1:** Chemical compositions of experimental feeds and treatment diets.

Experimental diets	DM %	% DM					
		OM	CP	NDF	ADF	ADL	TDN∗
Grass hay	91.28	84.32	7.39	61.38	41.15	9.25	51.46
Wheat bran	91.89	93.35	15.84	37.73	12.90	3.82	72.69
Sorghum grain	91.28	97.38	14.97	26.84	6.73	2.54	77.32
Molasses	74.50	91.00	3.80	0.20	0.00	0.00	82.38
Cotton seed cake	91.60	94.22	33.10	54.22	40.97	9.94	51.59
Noug seed cake	91.55	86.83	34.52	32.83	26.86	4.21	62.19
Sesame seed cake	90.73	89.28	41.45	21.11	8.73	2.19	75.82
Concentrate mixture							
T 1	91.44	92.66	20.25	33.93	17.85	4.67	68.97
T 2	90.89	92.50	20.66	32.43	15.59	5.79	70.66
T 3	90.52	90.89	20.84	28.35	13.45	2.84	76.03

ADF = acid detergent fiber; ADL = acid detergent lignin; CP = crude protein; DM = dry matter; NDF = neutral detergent fiber; TDN = Total digestible nutrient, ∗TDN = 82.38-(ADF∗0.7515) (NRC, 2001). T1 = *Ad libitum* grass hay +48% wheat bran (wb) + 15% molasses +35% cottonseed cake (csc) + 1% salt +1% limestone; T2 = *Ad libitum* grass hay +43% wb+ 20% sorghum grain +35% noug seed cake(nsc) + 1% salt +1% limestone; T3 = *Ad libitum* grass hay +47% wb + 16% molasses +35% sesame seed cake (ssc) + 1% salt +1% limestone.

### 2.3 Data collection procedures

#### 2.3.1 Growth performance and feed consumption

Feed offered and refused was recorded daily and daily feed intake was calculated as the differences between offer and refusal. Representative feed samples were collected and stored for each experimental animal and pooled over the feeding trial period for further chemical analysis. Each lamb was weighed at the beginning of the experimental period and every 10 days interval throughout the experiment after overnight fasting at 6:00 AM before daily feed offering to avoid feed effect. To obtained average daily gain (gram/day), initial live weight of the animal was subtracted from final live weight and then divided by the number of feeding days.

#### 2.3.2 Carcass traits

At the end of the experimental period, eighteen randomly selected lambs (six from each dietary treatment) were transported to the Abergelle International Export Abattoir, Mekelle, Ethiopia and rested for 3 days in covered yards to avoid stress. Prior to slaughter, lambs were fasted for 16 h from the feed with free access to water. Animals were slaughtered according to the standard commercial procedures after recording weight just before slaughter to obtain the slaughter body weight (SBW).

Skin, head, forefeet, hind feet and all the viscera and fat depots (scrotal, pelvic, kidney and gut fat) were removed to determine the hot carcass weight (HCW). Gut fat comprised omental and mesenteric fat. The dressing percentage (DP) was obtained as the proportion of HCW to SBW (DP = HCW∗100/SBW) as defined by Smith et al. (2015). Empty body weight was obtained by subtracting the weight of the digestive (stomach and intestine) contents from the slaughter weight.

#### 2.3.3 Meat sample preparation and quality determination

Carcasses were chilled to the cold room, with an average temperature of 4 °C, where they remained for 24 h hanging from hooks by the tendon of the gastrocnemius muscle. After carcasses were chilled, carcasses were taken from the cooling chambers and weighed again to determine the cold carcass weight (CCW) and chilling losses were determined as follows: Chilling losses (%) = [(Hot carcass weight - Cold carcass weight)/Hot carcass weight ]∗100. Rib-eye area (cm^2^) was measured by making a transverse section between the 13th thoracic and first lumbar vertebrae, thereby exposing the transverse section of the rib from the right half carcass after chilled at 4 °C overnight. The Rib-eye area was measured using a grid sheet for transparency tracing as described by Gomes Cunha et al. (2008). Subcutaneous fat thickness (mm) was determined using a digital caliper at 3/4 of the length from the medial side of the Longissimus thoracis et lumborum (LL) to its lateral side on the dorso-lumbar line. Afterward, LL samples from both right and left sides were removed from each chilled carcass between the 8^th^ and 12^th^ rib bone for sub-sectioning and further meat physicochemical analysis. The right LL was used for determination of pH, color, drip loss, cooking loss, and shear force while the left side samples were reserved for the determination of chemical composition. After taking the color measurements, the same steaks previously used for meat color measurements were vacuum packaged and aged for 7 days in chilling temperature (4 °C). After seven days of aging, steaks were placed in a frozen (-20 °C) until cooking loss and shear force analysis were performed. The day before the analyses, the frozen steaks of LL were thawed overnight at 4 °C. After thawing, steaks were subjected for subsequent measurement of cooking loss and shear force at the meat Laboratory unit of Hawassa University, Southern Ethiopia.

##### 2.3.3.1 Meat pH determination

The pH values of the LL were measured at approximately 24 h (pH24) *post-mortem* using a portable meat pH-meter (HANNA HI 99163, Hanna Instrument, Inc., Woonsocket, Rhode Island, USA)) having a sharp penetrating blade over the electrode, as described by AMSA (2012). The pH probe was cleaned with distilled water, calibrated with pH 4.1 and 7.1 buffer solutions between each measurement, and was inserted into the LL directly. Each sample was determined 3 times in different locations and the average value was obtained.

##### 2.3.3.2 Meat color determination

Meat color measurement was performed on steaks of LL muscle 24 h after chilling at 4 °C. Steaks were exposed on a flat surface of white background in the measuring room and allowed to air 30 min prior measurements. Spectrophotometer was set to the L∗ (lightness), a∗ (redness), and b∗ (yellowness) system using illuminate D65, an observer angle of 10°, with an aperture size of 5.0 mm. Values of L∗, a∗, and b∗ were recorded from three readings performed at different points on the surface of each steak, using a HunterLab MiniScan EZ spectrophotometer (Model 4500L; Hunter Associates Laboratory Inc.,Reston, VA) calibrated with black and white standardized plates between measurements (AMSA, 2012).

##### 2.3.3.3 Drip loss determination

To determine drip loss, the ‘EZ’ method following the protocol adapted by Logan et al. (2019) for alpaca was used. Briefly, a 60 g drip loss block was trimmed to 2.5 cm in length parallel to the muscle fiber using a purpose-built cylindrical (2.5 cm diameter) muscle corer. These samples were weighed and placed within specialized EZ drip loss containers. All samples were placed within a rack, in the same chiller for 48 h at an average temperature of 3.3 °C and average humidity of 83%. Subsequently, the residual drip was removed with a paper towel from samples, and these samples were separately reweighed to determine post drip weights. Drip loss was expressed as follows: Drip loss (%) = [(Initial weight - Final weight)/Initial weight ]∗100.

##### 2.3.3.4 Cooking loss determination

Steaks with 2.5 cm thickness were removed from each aged LL and weighed accurately (pre-cooked weigh), vacuum packaged and then frozen at -20 °C. The day before cooking, frozen steaks were thawed in refrigerator at 4 °C. Then the sample was immersed in a 72 °C water bath for 35 min in plastic bags, following which they were immediately cooled in cold tap water for 30 min. After cooling, samples were then removed from the bags, wiped with filter paper and then reweighed again (post-cooked weigh), calculated as the cooking loss (%) = [(pre-cooked weight – post-cooked weight)/pre-cooked ] ∗ 100 as described by (Hopkins et al., 2010).

##### 2.3.3.5 Meat tenderness (WBSF) determination

The cooked meat samples were then refrigerated overnight and were then subjected to the measurement of shear force using a GR texture analyzer (Tallgrass Solutions, Inc., Part No: GR-151, Kansas, USA) with a V-shaped Warner-Bratzler shear force, blade, operating at a constant speed of 225 mm/min. Shear force was calculated as the average of 6 subsamples (Hopkins et al., 2010). In brief, six cores (1.27 cm diameter, 2.5 cm length) were detached from each cooked sample along with the longitudinal orientation of muscle fiber. Then, the cores were sheared at right angles to the fiber long axis. Shear force data of each sample was recorded as means of the replicates. Cooked cores of the samples were placed in a V-shaped blade which shears through the core perpendicular to the muscle grain. The peak force (Newton) required to shear through the center of each core was recorded.

##### 2.3.3.6 Meat chemical composition determination

Evaluation of meat chemical composition (moisture, protein, intramuscular fat, and ash) was performed according to the methods described by the Association of Official Analytical Chemists (AOAC, 2005), at the Animal Science Nutrition Laboratory of Hawassa University, southern Ethiopia. Briefly, samples of 100 g obtained from the left LL of each lamb were trimmed for connective tissue and external fat. Thereafter, samples were dried by lyophilization, ground to 1 mm sieve size, and stored for subsequent chemical analysis. Ash content was determined by igniting meat samples at 600 °C for 8 h. Nitrogen content was determined by using the Kjeldahl method and protein was calculated as N×6.25. Fat was extracted by the Soxhlet method.

##### 2.3.3.7 Partial budget analysis

Partial budget analysis was done according the procedure of Upton (1979) to evaluate the economic advantage of the different treatment diets. In determining the partial budget analysis, variable costs and returns were involved in the calculation. Selling price difference of lambs in each treatment before and after the experiment was considered as total return (TR) in the analysis. Net return (NR) was calculated by subtracting total variable costs (TVC) from total returns (TR). Total variable costs include the costs of all inputs that change due to the change in production technology. The change in net return (ΔNR) was obtained by the difference between change in total return (ΔTR) and change in total variable cost (ΔTVC). This was used as a reference criterion for decision on the adoption of a new technology: ΔNR = ΔTR- ΔTVC. The marginal rate of return (MRR) measures the increase in net income (ΔNR) associated with each additional unit of expenditure (ΔTVC). This was expressed as: MRR (%) = ΔNR/ΔTVC X 100.

### 2.4 Statistical analysis

Data on carcass and meat qualities were subjected to analyses of variance (ANOVA) according to a randomized complete block design using the General Linear Models (GLM) procedure of SAS version 9.3 (SAS – Statistical Analysis Systems Institute Inc., 2010). Significant treatment means were separated using Tukey HSD at a 95% confidence interval.

## 3 Results

### 3.1 Growth performance

Table 4 presented final body weight, body weight change, average daily body weight gain (ADG) and feed conversion efficiency (FCE) of Begait lambs fattened under different feeding regimes. Final body weight and total weight gain were higher for lamb fed T3 than T1 and T2 (*P* < 0.05). Similarly, lambs supplemented with T3 ration had significantly (*P* < 0.001) higher ADG (158.6 g/day) than in T2 (120 g/day) and T1 (118 g/day). Lambs fattened under T3 had higher FCE (ADG: daily DM intake) than T1 and T2.

### 3.2 Carcass components

Slaughter performance is directly related to the economic importance of meat animals. The results of slaughter traits for Begait lambs kept under different feeding regimes are given in Table 2. Body weight (SBW), empty body weight (EBW), hot carcass weight (HCW), and cold carcass weight (CCW) was higher for lambs of T3 than in T1 and T2 (P < 0.001). Dressing percentage and chilling shrinkage were not affected by diets (*P* > 0.05).

**Table 2:** Slaughter performance of Begait lambs under different dietary treatments.

Parameter	T1	T2	T3	SEM	*P*-Value
Initial body weight (kg)	32.76	32.77	32.91	0.781	0.0780
Final body weight (kg)	42.22^b^	42.40^b^	45.60^a^	0.964	0.0374
Body weight gain (kg)	9.46^b^	9.63^b^	12.69^a^	7.369	0.0008
Average daily weight gain (g)	118.25^b^	120.37^b^	158.62^a^	2.806	0.0003
Dry matter intake (g/day/head)	1057.68^b^	1077.68^b^	1123.89^a^	6.036	0.0009
Total DM Intake (g/kg BW^0.75^)	63.987	64.907	64.242	0.453	0.0810
Dry matter intake (% body weight)	2.51	2.55	2.48	0.025	0.0871
Feed conversion efficiency	0.112^b^	0.112^b^	0.14^a^	0.007	0.0004
Slaughter weight (kg)	37. 00^b^	37.17^b^	40.68^a^	0.573	0.0007
Empty body weight (kg)	31.97^b^	32.40^b^	35.78^a^	0.537	0.0003
Hot carcass weight (kg)	17. 20^b^	17.43^b^	19.50^a^	0.280	0.0001
Chilled carcass weight (kg)	16.83^b^	17.02^b^	19.10^a^	0.278	0.0003
Chilling shrinkage (%)	2.13	2.39	2.05	0.107	0.0602
DPSW	46.47	47.35	47.98	2.806	0.0597
DPEB	53.80	54.11	54.52	0.515	0.0524
CCDP	45.48	46.21	46.99	0.533	0.0677
Rib-eye area (cm^2^)	13.38^b^	13.50^b^	16.13^a^	0.158	0.0002
Fat thickness (mm)	2.08^b^	3.50^b^	4.25^a^	0.376	0.0009

^a-b^ mean diet within a row not bearing common superscript are significantly different; SEM = Standard error of mean; LS = Level of significance; DPSW = Dressing percentage on slaughter weight basis; DPEB = Dressing percentage on empty body weight basis; CCDP = Cold carcass dressing percentage on slaughter weight basis.

Rib-eye area was influenced by the dietary treatments and higher for lambs receiving T3 (16.12 cm^2^) than that of lamb in T2 (13.50 cm^2^) and T1 (13.37cm^2^). Similarly, fat thickness showed significant difference (*P* < 0.001) among the diets (Table 2). Highest value was recorded in T3 (4.25 mm) followed by T2 (3.50 mm) and T1 (2.08 mm).

### 3.3 Meat quality characteristics

As shown in Table 3, all chemical composition parameters (moisture, protein and ash) except intramuscular fat (IMF) were not affected by diets (*P* > 0.05). IMF was significantly modulated by treatment rations (*P* < 0.05). Intramuscular fat (IMF) was higher in the LL from lambs fed on diets supplemented with T3 than those lambs supplemented with T2 and T1.

**Table 3:** Meat quality characteristics of LL of Begait lambs fed under different dietary treatments.

Parameter	T1	T2	T3	SEM	P- value
Moisture (% of fresh meat)	71.98	71.76	70.26	0.3177	0.078
Protein (% of fresh meat)	20.92	21.22	21.81	0.1857	0.063
Fat (% of fresh meat)	3.79^b^	3.87^b^	4.18^a^	0.0635	0.002
Ash (% of fresh meat)	1.96	1.88	1.86	0.0257	0.091
p^H^	5.56	5.47	5.43	0.056	0.059
L∗	29.43^b^	30.75^b^	33.97^a^	0.476	0.0008
a∗	10.99	9.99	10.84	0.222	0.088
b∗	9.38	8.46	8.75	0.506	0.066
Drip loss (%)	3.11	3.23	3.03	0.054	0.058
Cooking loss (%)	26.12^a^	24.39^b^	24.18^b^	0.204	0.034
Shear force (Newton)	42.63^a^	40.43^b^	40.67^b^	0.059	0.046

^a-b^ mean diet within a row not bearing common superscript are significantly different; SEM = Standard error of mean; L∗ Lightness and varies from 100 for perfect white to zero for black, a∗ measure redness when + ve, grey when zero, green when -ve; b∗ measure yellowness when + ve, grey when zero, blue when –ve; DM = Dry matter.

The technological meat quality characteristics of LL of Begait lambs fed under different treatments are presented in Table 3. Ultimate pH was comparable (*P* > 0.05) among the dietary groups. Coming to the meat color, L∗ in T3 (33.97) was recorded higher (*P* < 0.001), but no difference was observed between T1 (29.43) and T2 (30.75) (*P* > 0.05). Regarding the other two-color indexes (a∗ and b∗), there were quite similar (*P* > 0.05) among treatment groups, and a∗ values at 24 h ranged from 9.99 to 10.99. Dietary treatment had no significant effect (*P* > 0.05) on the drip loss percentage, which ranged between 3.03% and 3.32%. Cooking loss was significantly dictated by dietary treatments (*P* < 0.05). Meat sampled from sheep kept under T1 (26.12%) had higher cooking loss compared to meat sampled from sheep that received T2 (24.39%) and T3 (24.18%). Meat tenderness was significantly influenced by diets (*P* < 0.05). The measured WBSF (Newton) was significantly lower in T2 (40.40) and T3 (40.60) dietary groups compared to T1 (42.60) group diets. No significant difference (*P* > 0.05) was observed between T2 and T3 dietary groups in shear force (meat tenderness).

### 3.4 Partial budget analysis

The partial budget analysis of Begait lambs fed under different treatments are presented in Table 4. The difference in the net return and rate return among dietary treatment groups were due to the quality feed perform, better feed conversion efficiency, body weight gain, and difference in feed cost and selling price of the animals. The partial budget analysis also demonstrated that T3 had higher net return and rate return due to higher body weight gain (158.62 g/day) as compared to the other treatments that had relative body weight gain of 120.37 g/day and 118.25 g/day for T1 and T2 respectively. Therefore, T3 was better as compared to the other feeding options. However, the use of either diet depends on the availability and cost of feed ingredients.

**Table 4:** Partial budget analysis of Begait lambs fed under different dietary treatments.

Parameters	Treatments		
	T1	T2	T3
Purchase price	1965.60	1966.20	1974.60
Cost of basal diet	59.81	59.91	61.55
Cost of molasses	78.87	0.00	76.51
Cost of wheat bran	107.89	103.85	124.72
Cost of cotton seed cake	150.32	0.00	0.00
Cost of sorghum grain	0.00	73.77	0.00
Cost of noug seed cake	0.00	154.47	0.00
Cost of sesame seed cake	0.00	0.00	153.65
Cost of salt	2.10	2.21	2.35
Cost of limestone	0.42	0.44	0.46
Total variable cost	399.41	394.65	419.25
Δ TVC	0.00	4.76	24.60
Selling price of Sheep	3377.60	3392.00	3648.00
Total rate of return (TRR)	1412.00	1425.80	1673.40
ΔTRR	0.00	13.80	247.60
Net return (NR)	1012.59	1031.14	1254.15
ΔNR	0.00	18.55	223.01
Marginal rate of return (MRR %)	0.00	389.93	906.69

TVC = total variable cost; T_1_ = Hay *ad libitum* + concentrate mixture (48% wheat bran, 15% molasses, 35% cotton seed cake, 1% salt and 1% limestone); T_2_ = Hay *ad libitum* + concentrate mixture (43% wheat bran, 20% sorghum grain, 35% noug seed cake, 1% salt and 1% limestone); T_3_ = Hay *ad libitum* + concentrate mixture (47% wheat bran, 16% molasses, 35% sesame seed cake, 1% salt and 1% limestone).

## 4 Discussion

### 4.1 Growth performance

The higher DM intake observed in T3 in the present study could be associated with increased protein intake that contribute more nitrogen supply to the rumen microorganisms (Van Soest, 1994). This could lead to the fact that an increase in microbial population and efficiency, thereby facilitating the rate of breakdown of the digesta, which eventually leads to increment in feed intake. Subsequently, 38–40 g/day of daily body weight gain was higher in T3 as compared to lambs in T2 and T1. ADG (118–159 g) reported in the present study was higher than the results of 36–107 g/day for the same breed reported by different authors (Yirdaw et al., 2017; Gebrekidan et al., 2019; Haymanot et al., 2019). The experimental lambs in these previous reports achieved below their genetic potential for growth might be associated to restricted feed intake, which is much less than the requirement for growth (NRC, 1985; McDonald et al., 2002).

FCE value observed in this study was consistent with the trend of ADG for all treatments. In agreement to the present study, Pond et al. (1988) reported that diets that promote high rates of gain will usually result in greater efficiency than diets that do not allow rapid gain.

### 4.2 Carcass traits

Differences among treatments in hot carcass weight and rib eye area observed in this study were consistent with dressing percentage. The higher carcass weight recorded in T3 reflects that higher feed consumption enhanced muscle growth. Indeed, the recorded HCW of Begait lamb (17.20–19.50 kg) in the current study was superior to the entire carcass weight of Ethiopian indigenous sheep breeds reported previously in the literature by Ayele and Urge (2018). Notably, it was 95% elevated than that of the national average carcass weight (10 kg) of Ethiopian sheep (Legese and Fadiga, 2014) and 20–34% heavier than that reported for the same breed (Yirdaw et al., 2017; Gebrekidan et al., 2019; Haymanot et al., 2019), implying that the experimental lambs in those previous studies performed below their potential. The carcass weight was comparable with the average carcass weight (18.6 kg) of lambs in New Zealand (Ye et al., 2020). It was also as good as to the results of Mohammed and Yagoub (2016) who reported 17.20–18.00 kg for Sudan desert sheep. This implies that Begait lambs have faster growth rate and ability to reache at slaughter weight within short period of time.

Carcass chilling loss was not affected by diets. Chilling losses (2.13–2.39%) were lower than results 4.08–4.2% and 4.36–5.7% reported by Gashu et al. (2017) and Zewide et al. (2019) for Afar sheep and Washera sheep, respectively. Chilling losses ranging from 2.3 to 8.7% were noted for different goat breeds and weights (El Khidir et al., 1998; Getahun, 2001). These greater variations in chilling loss could be due to their differences in carcass fat content.

The higher rib-eye area of lambs supplemented with T3 group diet compared to T1 and T2 are attributed to the better plane of nutrition that probably led to better muscle production (Ayele et al., 2019). The values of the rib-eye area (13.36–16.13 cm^2^) obtained in this study were higher than the value (5.71–9.20 cm^2^) for Horro, Washera, Afar, and Arsi-Bale lambs reported by different researchers (Ayele et al.,2019; Zewide et al., 2019). The possible reason for the variation is due to the greater difference in slaughter weight, carcass weight, and dressing percentage. A positive correlation was seen between carcass weight and rib eye area in many scientific studies (Estifanos and Melaku, 2009; Hailu et al., 2011). The fat thickness value observed in this study was consistent with the trend observed for rib eye area across all treatments. Gashu et al. (2017) reported higher fat thickness and larger rib-eye area in sheep that consumed high supplement.

### 4.3 Meat quality characteristics

In the current study, fat contents of LL were influenced by diets, while moisture, protein and ash contents did not show so. It seems that the higher fat content value in T3 group diets could be an attribute of the heavier slaughter body weight of lambs exhibited higher fat deposition in the muscle, contributing to meat palatability characteristics such as juiciness, tenderness and flavor (Wood et al., 2008). The fat content of the meat obtained in this study was higher than the values of 2.61–2.62% reported by Gashu et al. (2017) for Washera sheep but was comparable to a recent target of 4–5% fat for lambs suggested by previous studies (Hopkins et al., 2006; Pannier et al.,2014). The observed contents of protein, moisture and ash were similar to those reported previously for the mutton of Ethiopian indigenous sheep breeds (Gashu et al., 2017; Zewide et al., 2019). Moreover, the moisture and protein values fell within the range of 70.80–80.30 and 18.5–23.4%, respectively depicted for values indicative of good quality meat (Bezerra et al., 2016; Abdallah et al., 2020).

Meat pH was not affected by diet and the ultimate pH values (5.43–5.56) were within the acceptable range for the export-import market, revealing the glycogen levels in the muscle of the meat was high enough to build up the optimum level of lactic acid causing a fall in pH and thereby improving the shelf life of the meat (Abebe et al., 2010). Hence, the optimum pH values measured in the present study showed that animals were in good physical condition with sufficient glycogen reserve at slaughter.

The higher L∗ value in T3 group diets could be due to the attribution of more intramuscular fat content that makes the meat luminous (Gashu et al., 2017). In agreement with this, Majdoub-Mathlouthi et al. (2013) and Zewide et al. (2019) pointed out that muscle from heavier slaughter body weight of lambs exhibited lighter (L∗) color than from those lighter lambs. The dietary treatment had no significant effect on the other two-color indexes (a∗ and b∗), in agreement with the findings of Gashu et al. (2017) and Zewide et al. (2019) in which a∗ and b∗ were not influenced by diet differences for Ethiopian sheep breeds. Since a bright red color is an important criterion for meat quality traits, influencing the purchasing decisions of consumers as it is the first factor seen by the consumer, and is used as an indication of freshness and wholesomeness (Joo et al., 2013; Ugurlu et al., 2017). Fresh lamb meat should have values higher than 34 for L∗ and 9.5 for a∗ to be considered acceptable by consumers. These values should rise to 14.5 and 44 for a∗ and L∗, respectively to satisfy 95% of the consumers (Khliji et al., 2010). With this thought, the meat of Begait lambs reared under T3 of L∗ was a better, whereas a∗ color indexes lie within the acceptable threshold.

In the present study, drip loss was not modulated by dietary treatment. This result did not agree with those of Oliván et al. (2001), who found an inverse relation between the fatness level and water-holding capacity. Logan et al. (2019) stated that drip loss is an indicator of water holding capacity in fresh meat that can be measured using the bag method. It is the water that is lost by meat because of the gravity force, which is an important quality criterion for the meat processing industry and the consumer (Fischer, 2007).

Cooking loss was affected by diets with higher for meat sampled from lambs fed on T1 as compared to meat from the other two diets. Differences in intramuscular fat content (Ueda et al., 2007) could partly explain the higher cooking losses in T3 dietary group. In our study, we found a negative correlation between cooking losses and intramuscular fat content. This tendency would agree with that reported by Ueda et al. (2007), who found a significant negative correlation between both the traits. Frank et al. (2016) also studied that an increasing intramuscular fat of beef from 5% to 22%, the water content decreased, but at the same time, the cooking loss also decreased. As reported by Lopes et al. (2014), the muscular structure could alter by intramuscular fat, allowing for the retention of higher levels of water, giving physical protection against muscle dehydration. Usually, cooking loss values from meat of small ruminants has falling within the range 14–41% (Ayeb et al., 2016).

Meat tenderness was significantly affected by diets and tender (less shear force) was recorded for meat samples from lambs fed in T3 as compared to lambs in diets T2 and T1. Meat with low shear force tends to have good tenderness and vice versa. The relatively less shear force of T3 could be attributed to a combination of factors that involve higher intramuscular fat and water holding capacity as explained by Kannan et al. (2006). In agreement with this, results obtained by Schönfeldt et al. (1993) indicate that fattened sheep and goat carcasses correlate positively with tenderness of muscle. It is believed that the consumer perceives cooked ruminant meat to be too tough if it has a shear force value of more than about 44–55 N (Ugurlu et al., 2017).

### 4.4 Partial budget analysis

The difference in the net return and rate return among dietary treatment groups were due to the quality feed perform, better feed conversion efficiency, body weight gain of the lambs, and difference in feed cost and selling price of the animals. The partial budget analysis also demonstrated that T3 had higher net return and rate return due to higher body weight gain (158.62 g/day) as compared to the other treatments that had relative body weight gain of 120.37 g/day and 118.25 g/day for T1 and T2 respectively. Therefore, T3 was better as compared to the other feeding options. However, the use of either diet depends on the availability and cost of feed ingredients.

## 5 Conclusions

The current study showed that growth, carcass and meat characteristics of fattening Begait lambs are affected by diet. A diet composed of 16% molasses, 47% wb, 35% ssc, 1% salt and 1% limestone gave better biological performance and economic return. This implies that feeding of Begait lamb with dietary treatment 3 is a better option for the production of heavy carcasses yield with superior quality of meat as compared to the other tested diets. However, the producer may choose one of the diets depending on the availability and cost of feed ingredients to maximize the performance of feedlot lambs. Future research is also warranted to explore the effects of this feeding regime on fatty acid composition and consumer acceptability meat of fattening Begait lambs to complement the results obtained in this study.

## Declarations

### Author contribution statement

Kahsu Atsbha: Performed the experiments; Analyzed and interpreted the data; Wrote the paper.

Tikabo Gebremariam: Conceived and designed the experiments; Wrote the paper.

Teferi Aregawi: Contributed reagents, materials, analysis tools or data; Wrote the paper.

### Funding statement

This work was supported by Tigray Agricultural Research Institute and Humera Agricultural Research Center.

### Data availability statement

Data associated with this study has been deposited at Kahsu.

### Declaration of interests statement

The authors declare no conflict of interest.

### Additional information

No additional information is available for this paper.
